# Who says we need to wait 30 minutes after the influenza vaccination?

**DOI:** 10.1002/jgf2.312

**Published:** 2020-03-16

**Authors:** Takashi Watari

**Affiliations:** ^1^ Postgraduate Clinical Training Center Shimane University Hospital Shimane Japan

To the editor

On one occasion, I accompanied my three children to the pediatric clinic for their influenza vaccinations. Such opportunities to go to the doctor as a patient or family member, rather than as a physician, always bring a new perspective. The waiting room was overflowing with children with fever, sneezing, and runny nose, and I was afraid of getting infected by them. The physicians instructed their patients to wait in the clinic for 30 minutes after receiving the influenza vaccine. We had to wait in a crowded clinic for more than an hour after three shots for the children—this was nothing special, as we may do the same in any institution in Japan. However, do we really need to wait 30 minutes after the influenza vaccination?

I conducted a literature review to investigate this question. I used Google Scholar and PubMed to search for keywords “influenza,” “vaccine,” and “anaphylaxis,” from January 27‐30, 2020. According to Japanese literature, the reason for having to wait for 30 minutes is anaphylaxis‐like symptoms, the most severe case of adverse reactions to the influenza vaccine. According to the Japanese Ministry of Health, Labour and Welfare, “shock and anaphylaxis‐like symptoms often occur relatively quickly after vaccination due to allergic reaction to the vaccine, so it is necessary to stay at the medical institution for 30 minutes after vaccination.”^1^ Therefore, medical institutions in Japan instruct their patients to rest for 30 minutes, assuming a potential for anaphylaxis after the influenza vaccination.

One previous study reported that among 2.52 million people administered vaccine (including 1.11 million administered influenza vaccine), 33 patients experienced vaccination‐driven anaphylaxis symptoms between January 2009 and December 2011 (Table 1).^2^ The diagnosis of anaphylaxis was made by the Brighton Collaboration Criteria,^3^ with only 1.3 out of every 1 million people having anaphylactic side reactions.^2^ There were no deaths, and only one patient was hospitalized among 2.52 million people. All cases received specific drug therapy, including epinephrine in 15 (45%), antihistamine in 28 (85%), and corticosteroid in 17 (52%), and only 5 (15%) cases needed intravenous therapy.^2^ However, only 24% of anaphylaxis occurred in less than 30 minutes, and more than half of the reactions occurred between 30 minutes and 4 hours after the vaccination. In other words, 76% of vaccine‐driven anaphylaxis cannot be prevented by simply resting in the facility for 30 minutes.^2^

**Table 1:** Time of anaphylaxis onset after the vaccination among 2.52 million people

Onset of anaphylaxis	Cases, n = 33 (%)
<30 min	8 (24%)
30 min to 2 h	8 (24%)
2‐4 h	10 (30%)
4‐20 h	3 (10%)
Unknown	4 (12%)

In Japan, where at least 17 million people receive influenza vaccines annually, a 30‐minute waiting period can be a cumulative waste of productive time nationwide, and the risk of droplet transmission in outpatient settings could be increased in the influenza season. Nevertheless, anaphylaxis can be a fatal disease, so it is undoubtedly very important to remain vigilant for symptoms after every influenza vaccination.

In anaphylaxis, which occurs in only 1.3 out of a million people and perhaps 25% of future patients within 30 minutes, there is probably little need to wait 30 minutes for every patient in a crowded environment. Moreover, 85% of the patients who developed anaphylaxis because of the vaccine had a history of atopic dermatitis. Hence, it may be much more beneficial for more high‐risk patients to receive a thorough explanation or pamphlet instruction to come to a medical institution immediately after showing symptoms of the vaccine side reaction, without waiting in a crowded place. I believe that generalists need to maintain flexible thinking to ask whether our practices are appropriate in cases of high uncertainty.

## CONFLICT OF INTEREST

The authors have stated explicitly that there are no conflicts of interest in connection with this article.
